# Pridopidine modifies disease phenotype in a SOD1 mouse model of amyotrophic lateral sclerosis

**DOI:** 10.1111/ejn.15608

**Published:** 2022-02-12

**Authors:** Héctor M. Estévez‐Silva, Tomás Mediavilla, Bruno Lima Giacobbo, Xijia Liu, Fahad R. Sultan, Daniel J. Marcellino

**Affiliations:** ^1^ Department of Integrative Medical Biology Umeå University Umeå Sweden; ^2^ Departamento de Ciencias Médicas Básicas, Instituto de Tecnologías Biomédicas (ITB) Universidad de La Laguna Santa Cruz de Tenerife Spain; ^3^ Umeå School of Business, Economics and Statistics Umeå University Umeå Sweden

**Keywords:** motor function, neuroprotection, preclinical research, pridopidine, sigma‐1 receptor, SOD1G93A

## Abstract

Amyotrophic lateral sclerosis (ALS) is a lethal and incurable neurodegenerative disease due to the loss of upper and lower motor neurons, which leads to muscle weakness, atrophy, and paralysis. Sigma‐1 receptor (*σ*‐1R) is a ligand‐operated protein that exhibits pro‐survival and anti‐apoptotic properties. In addition, mutations in its codifying gene are linked to development of juvenile ALS pointing to an important role in ALS. Here, we investigated the disease‐modifying effects of pridopidine, a *σ*‐1R agonist, using a delayed onset SOD1 G93A mouse model of ALS. Mice were administered a continuous release of pridopidine (3.0 mg/kg/day) for 4 weeks starting before the appearance of any sign of muscle weakness. Mice were monitored weekly and several behavioural tests were used to evaluate muscle strength, motor coordination and gait patterns. Pridopidine‐treated SOD1 G93A mice showed genotype‐specific effects with the prevention of cachexia. In addition, these effects exhibited significant improvement of motor behaviour 5 weeks after treatment ended. However, the survival of the animals was not extended. In summary, these results show that pridopidine can modify the disease phenotype of ALS‐associated cachexia and motor deficits in a SOD1 G93A mouse model.

## Graphical abstract

The insertion of the human SOD1 G93A mutation in transgenic mice progressively produces cachexia, paralysis, and finally, death resulting from the degeneration of motor neurons induced by the toxic gain‐of‐function of SOD1 protein. In our delayed‐onset SOD1 G93A mouse model, motor impairment becomes evident from 21 weeks of age, observed using different motor tests such as Pole Test, Inverted Screen Test, or Gait Test. The continuous administration of 3.0 mg/kg/day pridopidine for 4 weeks, using osmotic pumps, prevents cachexia and improves motor abilities 5 weeks after treatment withdrawal, pointing to a disease‐modifying effect of pridopidine.

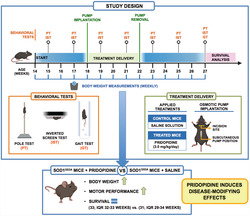

## 1 INTRODUCTION

Amyotrophic lateral sclerosis (ALS) is a neurodegenerative disease that affects both upper and lower motor neurons. Characterized by progressive motor deficits, ALS leads to muscle atrophy, paralysis, cachexia and eventual respiratory failure and death, 3 to 5 years after symptom onset. (Mulder et al., 1986; Robberecht & Philips, 2013) Only two compounds have been approved so far to treat ALS. (Hergesheimer et al., 2020) Nevertheless, these therapeutic strategies provide limited efficacy in modifying disease trajectory and further therapeutic approaches are required. (Petrov et al., 2017; Zoccolella et al., 2009)

Up to 10% of ALS are familial (fALS), (Dion et al., 2009) spanning over 30 different genes associated with either its onset or with disease progression. (Renton et al., 2014) Mutations in Cu/Zn superoxide dismutase (*SOD1*) represent one of the most common cause of fALS and results in up to 20% of cases, (Birve et al., 2010; Deng et al., 1993; Renton et al., 2014; Rosen et al., 1993; Rotunno et al., 2014) whereas mutations in *SOD1* are only found in around 1% of sporadic ALS (sALS). (Pasinelli & Brown, 2006) These genetic findings led to the development of the first transgenic mouse as an animal model of motor neuron disease, the G93A‐hSOD1 model. (Gurney et al., 1994) The validity of the SOD1 model was further corroborated by replicating many of the ALS symptoms found in humans. (Acevedo‐Arozena et al., 2011) This animal model has provided important insights into different disease mechanisms, addressing the role and contribution of oxidative stress, mitochondrial dysfunction, glutamate hyperexcitability, axonal transport dysfunction, protein misfolding and toxic aggregate formation to disease manifestation. (Hardiman et al., 2017) Numerous transgenic mouse models mimicking ALS symptoms have been developed over the past 25 years (for Review see (Hardiman et al., 2017)), with the SOD1 mouse model remaining the workhorse for evaluating basic mechanisms of neurodegeneration and therapeutic strategies.

Pridopidine (4‐[3‐(methylsulfonyl)phenyl]‐1‐propyl‐piperidine, formerly ACR16) is a small‐molecule investigational drug candidate that is currently in Phase II clinical trials for Parkinson's disease levodopa‐induced dyskinesia (PD‐LID) (McFarthing et al., 2019) and that has been announced to be one of five candidate compounds evaluated in a novel platform trial for ALS, the HEALEY ALS Platform Trial. (Sean, 2019) Prior to these trials, pridopidine has been evaluated in several trials for HD with varying results. (Johnston et al., 2019) While at high doses, pridopidine binds to multiple targets (Johnston et al., 2019) including dopamine D_2_ and D_3_, adrenergic α_2C_ and serotoninergic 5‐HT_1A_ receptors, at lower doses it binds with higher affinity to *σ*_1_ receptors (*σ*‐1R). (Sahlholm et al., 2013; Sahlholm et al., 2015) The concept of *σ*‐1R has evolved significantly over the past decades, in which today, it is clear that *σ*‐1R is not a traditional receptor, rather a non‐G‐protein coupled, non‐ionotropic intracellular chaperone at the endoplasmic reticulum (ER) that modulates Ca^2+^‐signalling. (Hayashi & Su, 2007; Kim, 2017; Sahlholm et al., 2015) Pridopidine positively influences *σ*‐1R‐regulated pathways across neurodegenerative and neurodevelopmental disorders that include protection against axonal and neuronal injury restoring spine impairments, enhancing BDNF secretion and restoring mitochondrial function. (Penke et al., 2018) The central nervous system (CNS) is especially rich in *σ*‐1R s and particularly within the lower motor neurons of the spinal cord. (Ryskamp et al., 2019) Although the precise causality of motor neuron degeneration in ALS remains largely unknown, (Mavlyutov et al., 2010) mutations in *σ*‐1R s are associated with fALS (Al‐Saif et al., 2011) and animal models of ALS that lack *σ*‐1R s present with exacerbated disease progression. (Mavlyutov et al., 2013) Furthermore, it was recently demonstrated that pridopidine improves several cellular and histological hallmark pathologies of ALS through *σ*‐1R s. (Ionescu et al., 2019a)

In this study, we evaluated the neuroprotective effect of a low dose (3 mg/kg/day) of pridopidine in a preclinical transgenic mouse model of motor neuron degeneration. We took advantage of hSOD1 transgenic mice with a lower number of transgene (G93A*), which results in a less aggressive progression of disease and allows for a prolonged monitoring of symptoms. Considering that development of human ALS may begin years or possibly decades prior to manifestation of symptoms and clinical diagnosis, (Eisen et al., 2014) early‐stage therapy could be key for ALS treatment. Therefore, we first determined the age of mice at which the earliest phenotype was detectable and subsequently initiated treatment prior to the appearance of any signs of muscle weakness. We investigated whether chronic pridopidine treatment was able to provide disease‐modifying effects by evaluating its influence on survival, weight loss and motor function‐related behaviours.

## 2 METHODS

### 2.1 Animals

In this study, the following transgenic models were used: hemizygous low copy number SOD1^G93A^ mice and hemizygous high copy number SOD1^G93A.1Gur/J^ mice (stock #: 004435, Jackson Laboratories, Bar Harbor, ME, USA). The mean ± S.D. lifespans of the mouse lines (in weeks) are 31 ± 3 (*n* = 12) and 21.4 ± 1 (*n* = 10), respectively. Low copy number SOD1^G93A^ (abbreviated here as G93A*) transgenic mice were provided by Thomas Brännström (Umeå University) in which observations from this particular colony revealed a delayed onset of ALS phenotype. (Lang et al., 2015) These mice were originally obtained from the high copy number SOD1^G93A.1Gur/J^ mice (stock #: 004435, Jackson Laboratories, Bar Harbor, ME, USA) and copy number reduction occurred during breeding of the colony. The transgene copy number was confirmed to be reduced in G93A* when compared with the founder line hSOD1^G93A.1Gur/J^ (abbreviated as G1H; Supp. Figure 1a,b), explaining the delayed onset of disease. Non‐transgenic (nTg) age‐ and sex‐matched littermates were used as controls (C57BL/6J). All animals were genotyped and the number of *hSOD1* copies was estimated as explained in detail below. Mice were randomized and housed in groups of 2–4 per cage under controlled humidity, temperature (23°C) and lighting (12‐h light/dark cycle). Food and water were provided ad libitum and mice were either euthanized at a preselected interval (28 weeks of age), or when mice reached humane endpoint. The humane endpoint was defined as the inability to right themselves within 5 s after being placed on their side. (Hatzipetros et al., 2015) Mice were checked daily for signs of paralysis and were weighed weekly. G1H were similarly bred on a C57BL/6J background to generate a high copy colony (25 ± 1.5 transgene copies) to estimate transgene copy number of the G93A* mice used in this study. (Alexander et al., 2004) A total of 86 G93A* animals were used in this study; 57 were used to evaluate body weight, 39 in behavioural tests of motor activity, 57 in gait analysis, 21 animals were sex‐matched and used to evaluate survival (for those details see Table S1). In addition, 14 mice of the G1H line were used to estimate transgene copy number for the G93A* line. All animal use and experimental procedures were performed in accordance with international guidelines (Percie du Sert et al., 2020) and procedures were approved by the Umeå Regional Ethics Committee for Animal Research (ethical permit: A17‐2019).

**FIGURE 1:**
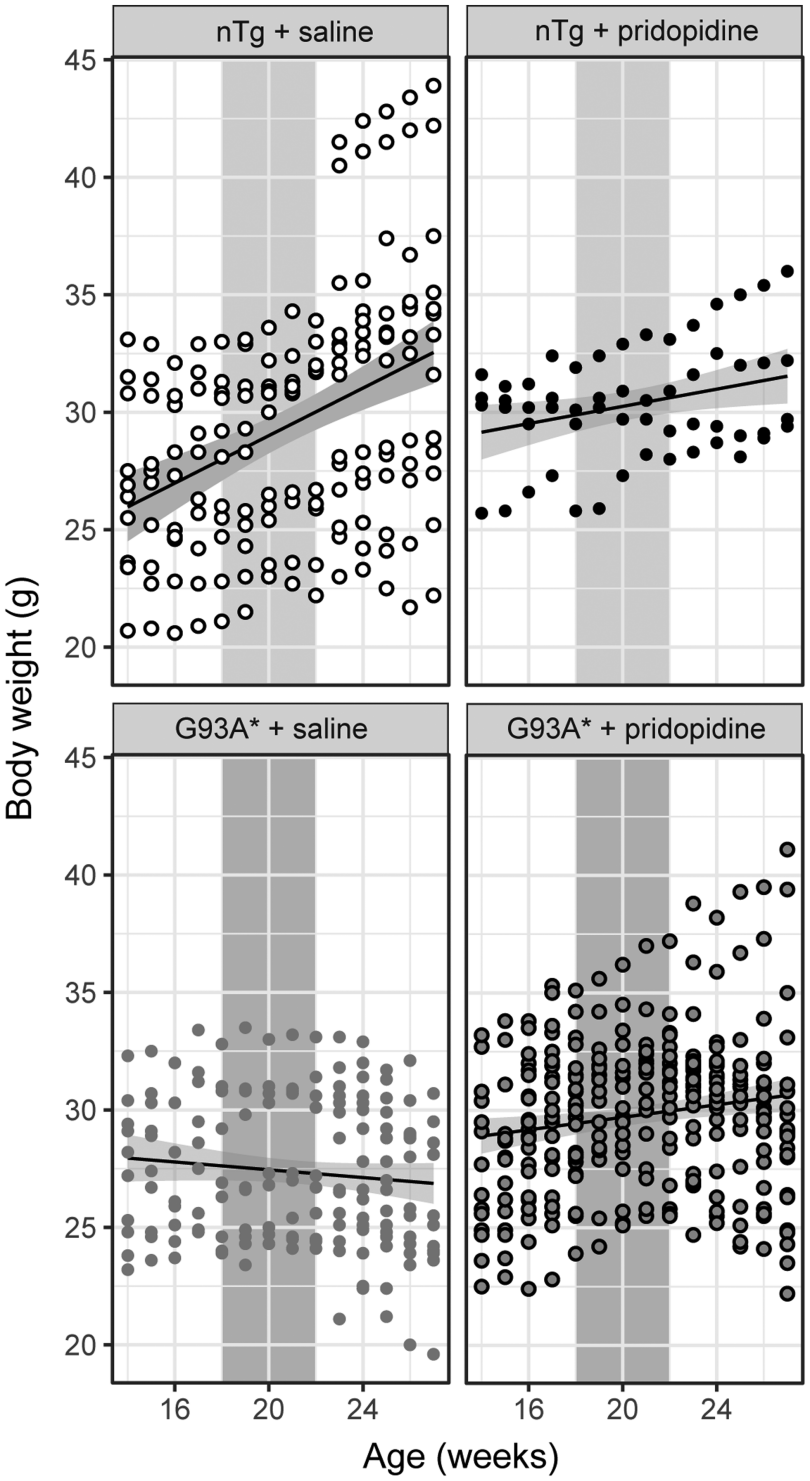
Pridopidine prevents weight loss in G93A* mice. Pridopidine hinders cachectic weight loss in G93A*. Animal weight was monitored before, during and after the treatment period (grey rectangle: 18–22 weeks). nTg littermates showed an increase in body weight (upper two panels) for both saline and pridopidine treated animals. Non‐treated G93A* mice showed a decline in body weight with time. Pridopidine prevented weight loss. Plotted is also regression line with 95% confidence intervals of slope and intercept (grey boundaries around fitted line). Weight data were further analysed with a linear mixed model and results are summarized in Table 1. Weight measurements were obtained from 14 nTg + saline, 4 nTg + pridopidine, 17 G93A* + saline and 22 G93A* + pridopidine animals

### 2.2 Genotyping and transgene copy number estimation

Initial genotyping of mice was performed by the Umeå Transgene Core Facility (UTCF) at the Umeå Centre for Comparative Biology (UCCB). Briefly, genomic DNA was extracted from earmarking biopsies and used as a template for PCR, using MyTaq™ Red Mix (Bioline Reagents Ltd., London, UK). The pairs of primers for *hSOD1* transgene genotyping were *hSOD1*F 5′‐CATCAGCCCTAATCCATCTGA‐3′, and *hSOD1*R 5′‐CGCGACTAACAATCA AAGTGA‐3′ (236 bp), and for murine interleukin‐2 precursor gene were: *Il2*F 5′‐CTAGGCCACAGAATTGAAAGATCT‐3′ and *Il2R* 5′‐GTAGGTGGAAATTC TAGCATCATCC‐3′ (324 bp). Murine interleukin‐2 precursor gene was used as a positive internal control, ensuring the correct functioning of the PCR reaction and distinguish transgenic mice from their wild‐type littermates. The cycling conditions were 96°C for 3 min, 9 cycles at 94°C for 45 s, 64°C for 1 min and 72°C for 2 min; followed by 24 cycles at 94°C for 45 s, 50.5°C for 1 min and 72°C for 2 min and a final step at 72°C for 10 min. PCR amplified products were resolved in 1.5% agarose stained with ethidium bromide and electrophoresed for 90 min under constant 110 V. Images were taken under a 365‐nm UV light lamp with a Bio‐Rad Gel Doc system and Bio‐Rad Image Lab 6.1.

Tail and/or ear tissue was collected for the subsequent isolation of genomic DNA (gDNA) using NucleoSpin Genomic DNA Tissue kit (Macherey‐Nagel, Düren, Germany). The amount of gDNA and its purity were determined using a NanoDrop 2000 spectrophotometer (Thermo Fisher Scientific, Waltham, MA, USA). A real‐time polymerase chain reaction (qPCR) was used to estimate *hSOD1* copy numbers in mice and in G93A* and in G1H mice. Real‐time qPCR experiments were performed using iTaq Universal SYBR® Green Supermix (Bio‐Rad, Hercules, CA, USA) in a thermal cycler C1000 (Bio‐Rad) connected to a CFX96 optical detector module (Bio‐Rad). Primer sequences for *hSOD1* were: *hSOD1*F 5′‐GGGAAGCTGTTGTCCCAAG‐3′ and *hSOD1*R 5′‐CAAGGGGAGGTAAAA GAGAGC‐3′ (88 bp) and for murine apolipoprotein B‐100 precursor gene were: *APO‐B100*F 5′‐CACGTGGGCTCCAGCATT‐3′ and *APO‐B100*R 5′‐TCACCAGTCATTTCTGCCTTTG‐3′ (74 bp). For each gene, all samples were measured in triplicate within the same 96‐well plate using the following cycling conditions: 95°C for 3 min and 40 cycles at 95°C for 10 s and 60°C for 30 s. Delta Cycle Threshold (ΔCt) was calculated for each sample as the difference between the internal control and the gene of interest Ct values. Individual *hSOD1* copy numbers were estimated based on the difference observed in ΔCt values of G93A* mice to the average value of ΔCt for G1H mice. (Lang et al., 2015) Since G1H mice carry 25 ± 1.5 copies of transgene (Alexander et al., 2004) and a difference of .5 in ΔCt value corresponds to a 33% reduction of transgene copy number (according to Jackson Laboratory guidelines), it was estimated that G93A* mice had an ~40% reduction in copy number. A ΔCt average value was obtained from all G93A* mice and an exclusion range was established by adding or subtracting .5 from the average, as half a cycle is the threshold for qPCR sensitivity for copy number changes. (Liu et al., 2003)

### 2.3 Pharmacological treatment

Male and female mice were randomized and divided into different groups: nTg controls, saline‐infused G93A* controls and pridopidine‐treated G93A* group (Table S1). We also had a fourth group of nTg pridopidine‐treated animals, which were mainly used in the weight measurement analysis. Saline and treatment was administered using Alzet® Osmotic Pumps (model 1004; DURECT Co., Cupertino, CA, USA) that were implanted subcutaneously. They were either filled with a saline solution (NaCl .9%; B. Braun, Melsungen, Germany) for control group, or with pridopidine (Axon Medchem, Groningen, Netherlands) dissolved in saline for the experimental group. Saline was degassed using argon flow prior to preparation of pridopidine and the filling and implantation of the osmotic pumps. These pumps provided a consistent and sustained release of saline or pridopidine at .11 μl/h for 4 weeks after which the pumps were removed 5 weeks after their surgical implantation. Administration of pridopidine was 3.0 mg/kg/day.

### 2.4 Behavioural tests

Multiple behavioural tests were performed to evaluate grip strength, motor coordination, balance, and gait patterns. Experimenters were blind in all instances to both genotype and treatment regimen. Both the inverted screen test and the pole test were performed and video recorded every other week from 16 weeks of age, while a gait analysis, using the footprint test, was conducted only once at 27 weeks of age.

#### 2.4.1 Kondziela's inverted screen test

To evaluate animal motor performance, coordination and muscular strength, individual mice were placed in the centre of a framed 50 × 50 cm metal wire screen with a 10 × 10 mm square mesh. This square metal wire screen was suspended 50 cm over a cushioned surface and rotated 180°, to position the mouse upside down grasping onto the screen. Once the screen is inverted, the time each animal remained suspended/hanging to the wires was timed. Three consecutive trials for a total of 120 s, with a 120 s inter‐trial interval, were recorded during three consecutive testing days. The latency to fall was calculated from the nine trials recorded.

#### 2.4.2 Pole test

The pole test was used to evaluate motor coordination, based on the ability of mice to grasp and manoeuvre on a pole in order to descend to its home cage. Mice were trained to complete the pole task over two consecutive days, prior to video recording their performance on the third day. A vertical wooden pole (50 cm long and .8 cm of diameter) was placed in the home cage. During training, the mice were placed with their head oriented upward on top of the pole for five trials, with a 60‐s inter‐trial interval. Mice will naturally orient themselves downward and descend the length of the pole in order to return to their home cage. Their performance is evaluated on the third day through three trials. The time spent to orient themselves downward (T‐*turn*) and the total time to descend to the base (T‐*total*), were timed. A time cut‐off of 8 s for T‐*turn* and 12 s for T‐*total* was used as the maximum time value in case the animal failed to complete the manoeuvre required for each task.

#### 2.4.3 Gait analysis

Immediately prior to the test, animals were trained to walk through a brightly lit narrow alley (to assist walking in a straight line), which led to a dark box at the end of the alley. Gait performance was evaluated at 27 weeks of age 5 weeks after treatment ended. After 2–3 test trials, the paws were painted blue (fore paws) or orange (hind paws) using coloured non‐toxic tempera paint, before allowing mice to walk the 50‐cm‐long and 8‐cm‐wide corridor lined with absorbent paper. A total of three gait trials were recorded and the paws were repainted before each new trial. The absorbent papers were dried, scanned and the footprint traces were analysed using Fiji‐ImageJ. The trial where the mouse showed the least deviation from a straight path without stopping or turning was chosen. Three gait cycles were measured from sequential steps of good quality footprints where parts of the foot were clearly identifiable (Brooks et al., 2012) and evaluated by measuring the stride length for both fore and hind limb, measuring the distance between the right and left side footprints in hind‐ and forelimbs (base width) and the distance between fore and hind limb paws (termed overlap). (Brooks et al., 2012) The trial that showed the least deviation from a straight path was chosen (usually the first and was termed best trial) and the average value of the three cycles was taken. The experimenter was not aware of the animals ID (and thereby treatment status) before and during behavioural experiment and only uncovered this at the end to document its ID on the absorbent paper. We also included a subsequent analysis using all nine gait cycles to compare parameters of individual gait cycles.

### 2.5 Statistical analyses

Effects of treatment on body weight was analysed with linear mixed models (lmm) using the lme4 (Bates et al., 2015) including fixed factors (age, sex, genotype and treatment), random factors (individual mice) and their interactions (Age: Treatment, Age:Sex, Genotype:Treatment):

$$\begin{array}{c} {\mathrm{BW}}_{\mathrm{ij}}=\beta_{0}+\beta_{1}A_{\mathrm{ij}}+\beta_{2}S_{\mathrm{ij}}+\beta_{3}A_{\mathrm{ij}}T_{\mathrm{ij}}+\beta_{4}A_{\mathrm{ij}}S_{\mathrm{ij}}+\\ \;\beta_{5}A_{\mathrm{ij}}T_{\mathrm{ij}}+\beta_{6}{\mathrm{Gt}}_{\mathrm{ij}}T_{\mathrm{ij}}+b_{0j}+b_{1j}A_{\mathrm{ij}}+\epsilon_{\mathrm{ij}} \end{array},$$

where *BW* is body weight, *A* is age, *S* is sex, *T* is treatment, *Gt* is genotype, the indices of observation i = 1,2,3, …, 679, and the indices of group factor j = 1,2,3, …, 57. For the random part, we assume that the random noise ε_ij_ ~ *N*(0, *σ* ^2^), the random effect *b*_0j_ *~ N*(0, *σ*_0_^2^) and random effect *b*_1j_ *~ N*(0, *σ*_1_^2^). We also assume that all random components are mutually independent. BW as the predicted model outcome was tested for normal distribution with the Shapiro‐test (package lme4 in R) and the data were power transformed by transformTukey (package rcompanion in R) to ensure normal distribution. In our model we applied random effects on the intercept and on the slope term of age within each individual. Factors and their interaction were assessed with the Wald *χ* ^2^ value and corresponding *p* value. Multiple contrasts were performed and p values were corrected for multiple comparison with the Holm‐Bonferroni method. The factor pridopidine treatment was tested in two different ways: treatment only during period of pump implant (weeks 18 to 22) and treatment for the whole duration after implant (weeks 18 to 27). Finally, we also tested whether including an additional Sex:Treatment interaction would yield an improved model but found no improvement by testing the two models with an Anova *F* test.

The longitudinal analysis of the behavioural tasks inverted screen test and pole test required a special statistical analysis, due to the fact that the dependent variable of test performance is highly skewed. Termination of the inverted screen test after 120 s of successful hanging led to skewed results and applying a maximum time cut‐off to complete the maneuvers of the pole test of 8 s for T‐*turn* and 12 s for T‐*total* also led to highly skewed responses. Therefore, a Cox regression analysis was performed using the coxme R‐package (Therneau et al., 2003) with the response as a time length variable (time‐to‐event), using a survival fit with fixed effects (age, sex and treatment), interactions (age: treatment) and random effects (intercept for individual mice and slope for age).

$$\begin{array}{rcl} \lambda \left(t\right) & = & \lambda_{0}\left(t\right)\exp\{\beta_{0}+\beta_{1}A_{\mathrm{ij}}+\beta_{2}S_{\mathrm{ij}}+\beta_{3}T_{\mathrm{ij}}+\beta_{4}A_{\mathrm{ij}}\\ & & \times T_{\mathrm{ij}}+b_{0j}+b_{1j}A_{\mathrm{ij}}+\epsilon_{\mathrm{ij}}\}, \end{array}$$

where $\lambda_{0}\left(t\right)$ is baseline hazard function, the indices of observation *i* = 1, 2, 3, …, 168, and the indices of group factor *j* = 1, 2, 3, …, 24. The log of hazard ratio is modelled by the linear predictor with fixed and random effects. For the random part, we assume that the random noise ε_ij_ ~ *N*(0, *σ* ^2^), the random effect *b*_0j_ *~ N*(0, *σ*_0_^2^) and random effect *b*_1j_ *~ N*(0, *σ*_1_^2^). We also assume that all random components are mutually independent. Data comparison was limited to treated and untreated G93A* and the effect of body weight was added and tested for in a second Cox‐regression analysis.

Effects of pridopidine treatment on gait performance were assessed with a general linear model (GLM) testing gait parameters (single values per animal obtained from averaged ‘best’ trial) for fore and hind paw overlap, fore and hind limb stride length and fore and hind limb base width as response and observing the effects of sex, genotype, treatment or the interaction genotype and treatment as dependent variables. Subsequently, we compared the data within a factor as pairwise comparisons and the tests were corrected for multiple comparisons using Bonferroni correction. We also assessed the repeated trials and measurements (a total of nine gait cycle analysis per animals) to compare parameters of individual gait cycles. This analysis was conducted by comparing the overlap fore paw and hind paw of each side with the stride length of the same side. The repeated measure regression was performed with a generalized estimating equations (‘geeglm’ from the ‘geepack’ library in R) with individual mice as repeated measure and for each genotype and treatment separately.

Data were either expressed as the mean or median ± S.E.M.; or by presenting individual values. Survival was estimated using the Log‐rank (Mantel–Cox) analysis. The significance level to reject null hypothesis was set at a *p* value of <.05.

## 3 RESULTS

### 3.1 Treatment with pridopidine prevents transgene‐associated weight loss in G93A*

SOD1^G93A^ transgenic models of ALS typically exhibit weight loss and this weight loss was significantly reduced in pridopidine treated G93A* mice. As depicted in Figure 1, body weight was monitored each week starting at 14 weeks, 4 weeks prior to the surgical implantation of osmotic pumps at 18 weeks of age. A gradual weight loss was observed in non‐treated transgenic mice as opposed to nTg littermates, which started around 21 weeks of age and continued to decrease throughout the entire study. In contrast, pridopidine prevented weight loss in G93A* mice. This was statistically evaluated with a lmm model with age, sex, genotype and treatment as fixed effects and included the fixed interactions treatment and genotype, age and treatment and age and sex. The intercept and age weight gains was included as random effects for the individual mice. The largest fixed effect was sex, followed by the interaction treatment and genotype (see Table 1). Sex weight effects included the heavier nTg males and the effect of genotype and treatment. Including the sex and treatment interaction did not show any significant improvement of the model (*χ* ^2^ = .006, *p* = .94).

**TABLE 1:** Reports of the linear mixed‐effects models for fixed factors and their interactions (A) and multiple comparisons test (B) with different treatment duration effects for body weight

Part (A)		Treatment duration model			
		4‐week treatment		Entire duration	
Factors and interactions	df	*χ* ^2^	*p* value	*χ* ^2^	*p* value
Age	1	4.30	<.05	.61	.44
Sex	1	77.90	<.001	81.33	<.001
Treatment × Genotype	3	30.78	<.001	49.10	<.001
Age × Treatment	1	9.64	<.01	21.00	<.001
Age × Sex	1	7.23	<.01	6.00	<.05
Marginal *R* ^2^/Conditional *R* ^2^		.31/.972		.304/.971	

Part (B) Tukey's multiple comparison test				
	4‐week treatment		Entire duration	
Contrast	*z* value	*p* value	*z* value	*p* value
nTg + pridopidine versus G93A* + pridopidine	−3.73	<.001	−3.64	<.001
G93A* + saline versus G93A* + pridopidine	2.19	<.05	−5.81	<.001
nTg + saline versus G93A* + pridopidine	.43	.670	−6.50	<.001
G93A* + saline versus nTg + pridopidine	4.05	<.001	−1.60	.110
nTg + saline versus nTg + pridopidine	3.20	<.01	−4.18	<.001
nTg + saline versus G93A* + saline	−2.52	<.05	−2.66	<.05

*Note*: Sample size: nTg + saline (*n* = 14); nTg + pridopidine (*n* = 4); G93A* + saline (n = 18); G93A* + pridopidine (*n* = 26).

We further explored the long‐lasting effect of treatment by testing two models with different treatment duration/effectiveness. We tested whether pridopidine treatment affected body weight during the 4**‐**week application period only, or whether it had a longer and disease modifying effect influencing body weight the entire duration of the observation period (up to 27 weeks). We observed a larger *χ* ^2^ value for the entire duration (*χ* ^2^ = 49.1, df = 3, *p* < .001) pointing to a disease modifying effect. The amount of variance explained by our models was very similar (see Table 1) with the marginal *R* ^2^ explaining 1/3 of the variability and the conditional explaining 97%.

A post hoc test with multiple comparison showed that pridopidine treatment modified body weight between the treated and untreated G93A* (Table 1). The largest gain in body weight of the treated versus untreated G93A* was observed with the extended duration model. However, this long‐term gain in body weight in the treated G93A* did not approach that of the nTg. We also tested the influence of ΔCt on body weight and found a significant interaction between ΔCt and treatment (see Figure S1c).

### 3.2 Monitoring motor performance during pridopidine treatment

**FIGURE 2:**
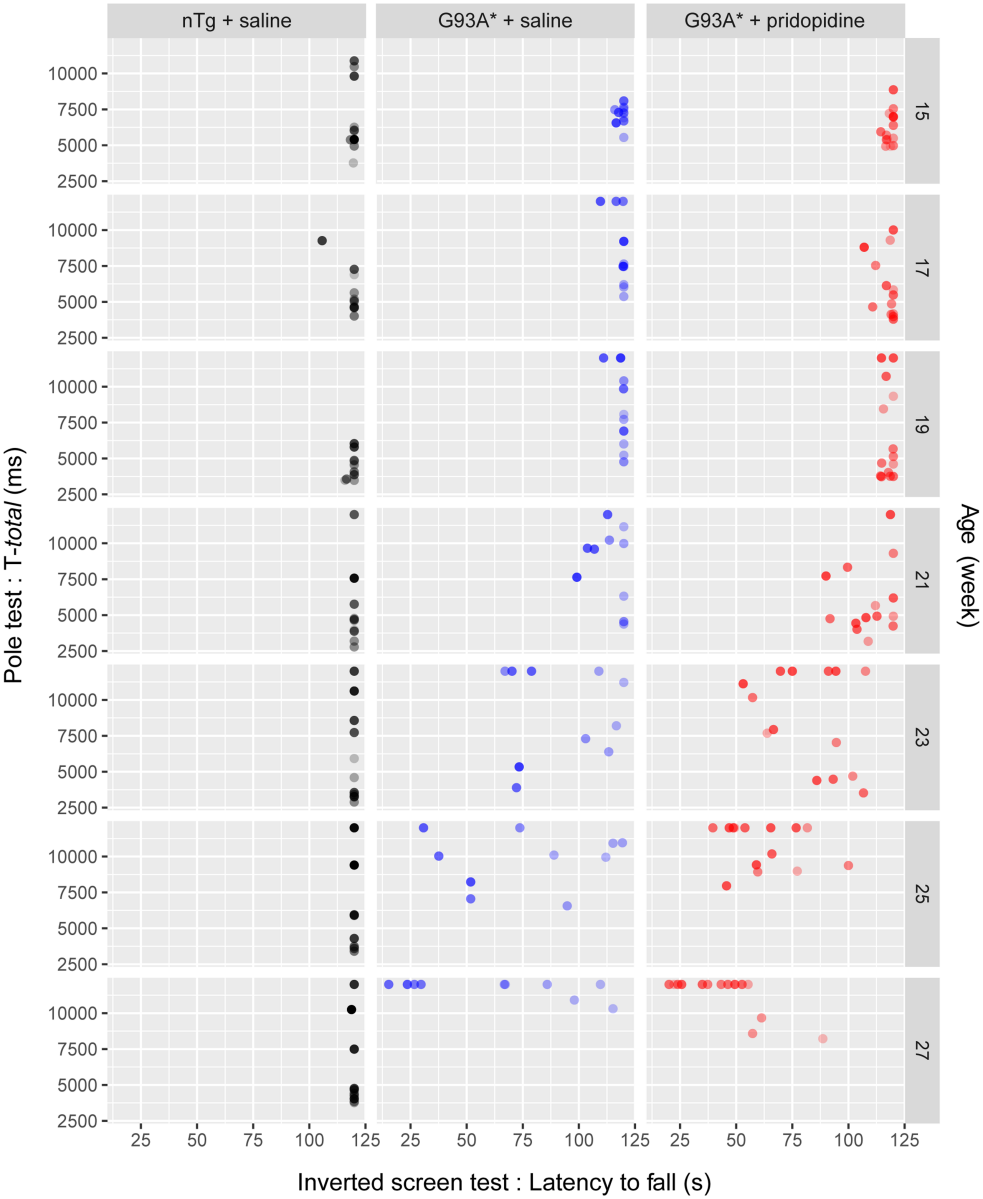
Motor performance monitoring of pridopidine treatment of G93A* phenotype development. Scatter plot of two motor tests are displayed. The inverted screen test (*x* axis) allows for a clear separation of nTg from SOD1 mice (shorter times indicate worse performance and inability to hang onto the screen). The difference becomes evident after 21 weeks. The *T*‐total in the pole test measures the time it takes mice to climb down the pole and is capped at 12 s. Animal weight is encoded with different transparencies (○ ≤ 25 g, ● 25 < *x* ≤ 30 g and ● ≤ 35 g) and shows that in untreated G93A* mice, animals weighting less performed better in the wire hanging test. In contrast treated G93A* mice performed worse in the inverted screen test but displayed better pole test performance during the treatment period (around week 21). Behavioural data were obtained from 11 nTg + saline, 10 G93A* + saline and 14 G93A* + pridopidine animals

G93A* mice characteristically show a progressive loss of grip strength due to the deterioration of the neuromuscular junction. (Harandi, 2016) We used the inverted screen test to monitor grip strength. G93A* mice failed to maintain their grip and fell earlier from the screen than nTg animals (Figure 2). This weakness in grip strength is observed earlier in male than in female G93A* mice (21 and 23 weeks of age, respectively). Pridopidine failed to prevent the loss of grip strength in G93A* treated mice, which had very similar latencies to fall from the inverted screen as G93A* control mice (Figure 2).

**FIGURE 3:**
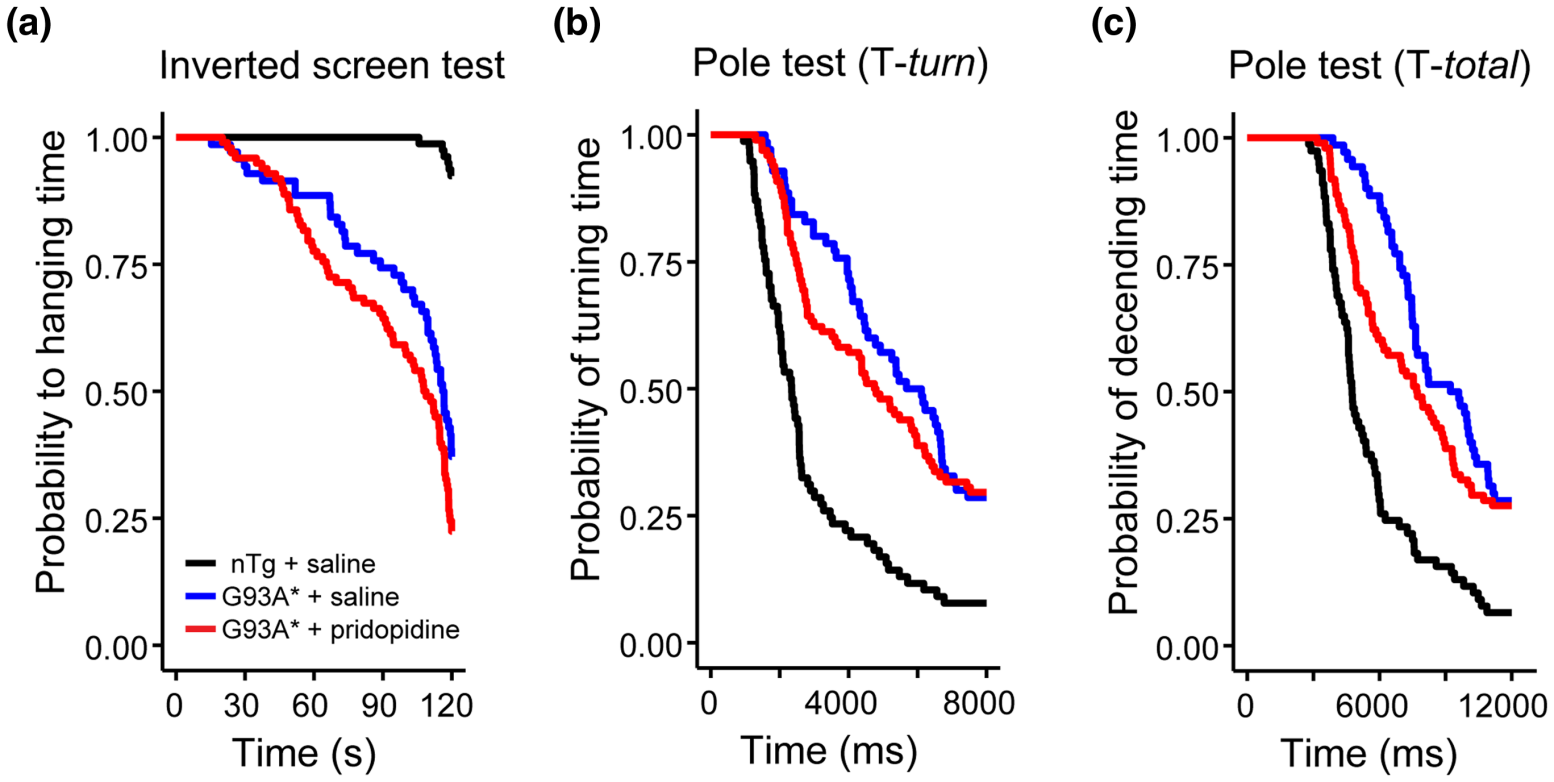
Time‐to‐event analysis of inverted screen and pole test. Animal performance is plotted as time‐to‐event. In the inverted screen, time‐to‐event is reached when the animal loses the grip to the wire and falls (a), whereas, in the pole test, the event occurs when the animal either turns around on the pole tip or manages to climb down the pole (b, c). For comparison we plotted nTg (*n* = 10) together with G93A* treated (*n* = 10) and untreated (*n* = 14) animals. Pridopidine treatment had different effects on motor outcome. In the case of the inverted screen test, it worsened animal behaviour, while in the pole test we observed an improvement. Behavioural data were obtained from same G93A* + saline and G93A* + pridopidine animals as in Figure 2

Deficits in motor behaviour associated with *hSOD1* transgene are detected earlier using the Pole Test than in the Inverted Screen Test, (Mediavilla et al., 2020) pointing to the Pole Test as a more sensitive test for early‐stage treatment studies. Comparing both tests (Figure 2) the G93A* took longer to climb down the pole after week 17, which appeared delayed for the pridopidine treated animals to week 21. To test whether both motor behaviour show any difference related to the pridopidine treatment we conducted a Cox regression for time‐to‐event, with the event being either time to turn/descent from the pole or time to hang on the inverted screen test (Figure 3 and Table 2). As expected, age of the animal had a strong effect on animal performance in all tests, leading to reduced latency to fall (Figure 3a) and increased time required to descend in the pole test (Figure 3b,c). Inclusion of animal weight as additional factor did not show significance for this factor (Table 2). G93A* males performed significantly worse in both the inverted screen and pole test. In summary, pridopidine treatment had different effects; while it improved performance in the Pole test leading to reduced time to descend the pole, the inverted screen test showed worse performance of pridopidine treated animals.

**TABLE 2:** Cox regression mixed‐effects for factors and interactions in motor test performance

Results of Cox regression analysis								
	Model 1				Model 2			
Factors and interactions	Coef.	s.e	z	*p*	Coef.	s.e	z	*p*
**Inverted screen test:** Latency to fall (G93A* only)								
Age	.753	2.123	10.440	<.001	.753	2.124	10.460	<.001
Sex (males)	1.682	5.374	2.870	<.01	1.724	5.608	2.580	<.01
Treatment (saline)	−1.311	.269	−.690	.490	−1.251	.286	−.650	.520
Age × Treatment (saline)	−.003	.997	−.040	.970	−.016	.984	−.170	.870
Animal weight					−.007	.993	−.080	.940
**Pole test:** Time to turn (G93A* only)								
Age	−.271	.763	−7.820	<.001	−.263	.769	−7.420	<.001
Sex (males)	−.828	.437	−2.590	<.01	−.610	.543	−1.510	.130
Treatment (saline)	−1.912	.148	−1.890	.059	−1.665	.189	−1.580	.110
Age × Treatment (saline)	.082	1.085	1.670	.095	.066	1.068	1.280	.200
Animal weight					−.055	.947	−.830	.400
Pole test: Total time to descend (G93A* only)								
Age	−.258	.772	−7.780	<.001	−.251	.778	−7.450	<.001
Sex (males)	−.754	.470	−2.840	<.01	−.588	.555	−1.700	.089
Treatment (saline)	−2.260	.104	−2.380	<.05	−2.064	.127	−2.110	<.05
Age x treatment (saline)	.090	1.094	1.880	.060	.078	1.081	1.560	.120
Animal weight					−.040	.961	−.700	.480

*Note*: Model 1: Age + **Sex** + Treatment + Age x Treatment + (1 + Age|ID). Model 2: Age + **Sex** + Treatment + Animal weight + Age x Treatment + (1 + Age|ID). Sample size: nTg + saline (*n* = 11); nTg + pridopidine (*n* = 4); G93A* + saline (*n* = 10); G93A* + pridopidine (*n* = 14).

### 3.3 Pridopidine treatment has a lasting effect on reducing gait abnormalities in SOD1G93A* mice

Footprint pattern analysis is commonly used to reveal gait abnormalities associated with altered motor coordination and balance due to diseases of the motor and vestibular systems. (Carter & Shieh, 2015) G93A* mice show their symptomatic gait around 27 weeks. The gait deficits show decreased hind limb base width and increased distance between hind limb paws with the fore limb paws (termed overlap). The increased distance is due to a decrease in hind limb and fore limb stance length. The weakened hind limb musculature makes it difficult for the animals to sufficiently advance the limb and also requires the hind limb to be placed under the body to minimize gait instability.

**FIGURE 4:**
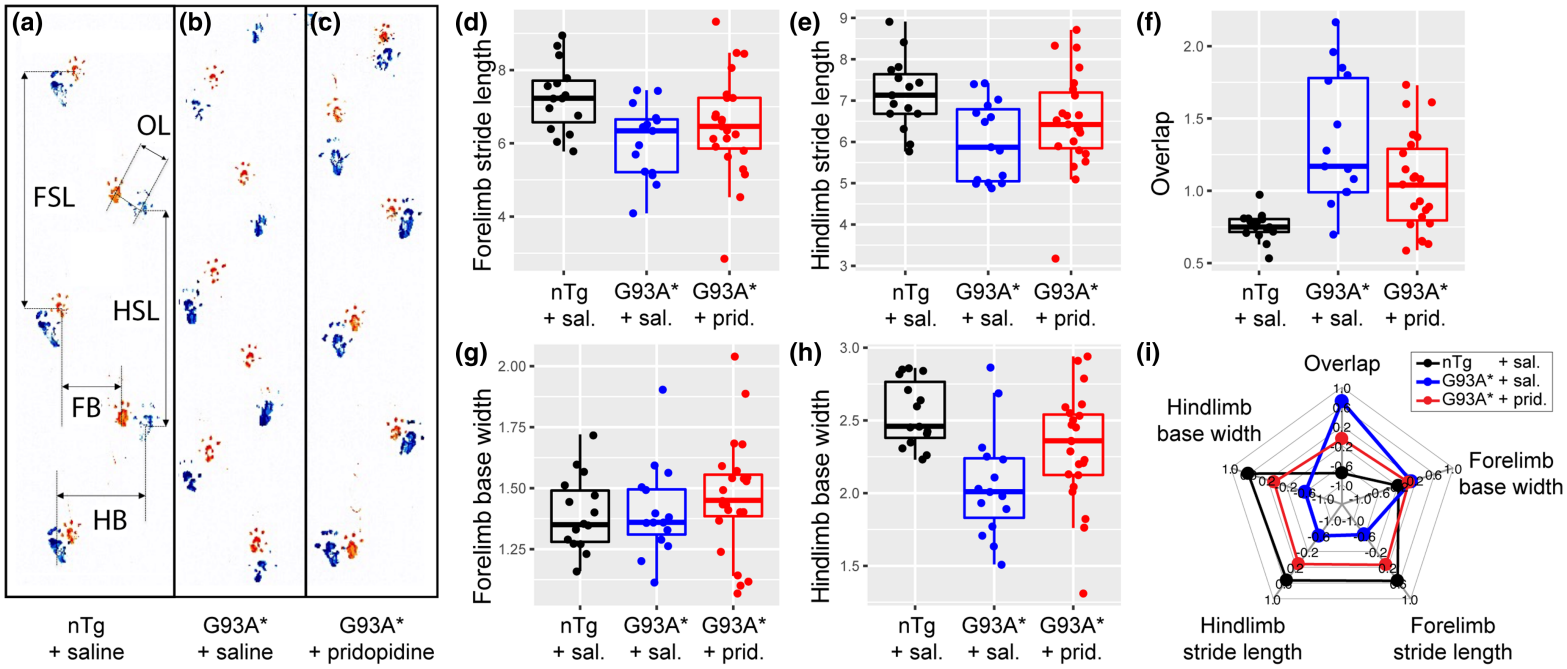
Gait analysis of nTg, treated and untreated G93A* mice. Gait footprints were quantified by measuring forelimb base width (FB), hindlimb base width (HB), fore and hindlimb stride length (FSL, HSL, respectively) and the distance between fore and hind paw prints (termed overlap, OL). Gait trials were repeated three times and the clearest prints were selected for analysis. (a–c) Paw prints for nTg, G93A* untreated and treated animals, respectively. (d–h) Boxplots of the different gait parameters with boxes with 25th, median and 75th percentiles and whiskers mark either max/min value or 1.5* interquartile range. In most gait parameters pridopidine treatment reverses the pathological G93A* pattern (except for forelimb base). This is summarized in the spider plot (i), where all five parameters are plotted as *z* score. GLM analysis showed a significant effect of treatment in gait parameter overlap (genotype treatment interaction Wald *χ* ^2^ = 43; post hoc test treated versus untreated G93A* mice *p* < .05). Data analysed are from nTg (*n* = 15); G93A* + pridopidine (*n* = 23) and G93A* + saline (*n* = 15) animals

Gait parameters were first quantified by choosing a single best stride cycle and quantifying the five gait parameters. This analysis confirmed that footprints of untreated G93A* mice had significantly different gait parameters than nTg mice in four of the five measurements with the exception of fore limb base width (Figure 4 and Table 3). Pridopidine was found to improve motor coordination (Figure 4) in transgenic mice and resulted in a significant improvement of footprint overlap compared to untreated G93A* mice (GLM with interaction genotype and treatment *χ* ^2^[2,50] = 42.97, *p* < .001 and post hoc test treated vs. untreated G93A* *p* < .05). Most of the other parameters also showed improvement and were similar to the nTG pattern (Figure 4i), however, they did not reach significance.

**TABLE 3:** GLM analysis of gait patterns: Post hoc tests and interactions

Overlapping						
	nTg + saline		G93A* + saline		Interaction sig.	
	Mean difference	*p* value	Mean difference	*p* value	Wald *χ* ^2^	Sig.
nTg + saline	—	—	—	—	42.968	<.001
G93A* + saline	−.599	<.001	—	—		
G93A* + pridopidine	−.287	<.001	.310	<.05		

Forelimb stride length						
	nTg + saline		G93A* + saline		Interaction sig.	
	Mean difference	*p* value	Mean difference	*p* value	Wald *χ* ^2^	Sig.
nTg + saline	—	—	—	—	13.764	<.01
G93A* + saline	1.421	<.01	—	—		
G93A* + pridopidine	.686	.190	−.735	.231		

Hindlimb stride length						
	nTg + saline		G93A* + saline		Interaction sig.	
	Mean difference	*p* value	Mean difference	*p* value	Wald *χ* ^2^	Sig.
nTg + saline	—	—	—	—	13.814	<.01
G93A* + saline	1.368	<.001	—	—		
G93A* + pridopidine	.626	.334	−.741	.159		

Forelimb base width						
	nTg + saline		G93A* + saline		Interaction sig.	
	Mean difference	*p* value	Mean difference	*p* value	Wald *χ* ^2^	Sig.
nTg + saline	—	—	—	—	1.258	.533
G93A* + saline	−.067	1.00	—	—		
G93A* + pridopidine	−.063	.967	.004	1.00		

Hindlimb base width						
	nTg + saline		G93A* + saline		Interaction sig.	
	Mean difference	*p* value	Mean difference	*p* value	Wald *χ* ^2^	Sig.
nTg + saline	—	—	—	—	21.849	<.001
G93A* + saline	.471	<.001	—	—		
G93A* + pridopidine	.215	.074	−.256	.077		

*Note*: Sample size: nTg + saline (*n* = 15); G93A* + saline (*n* = 15) and G93A* + saline (*n* = 23).

**FIGURE 5:**
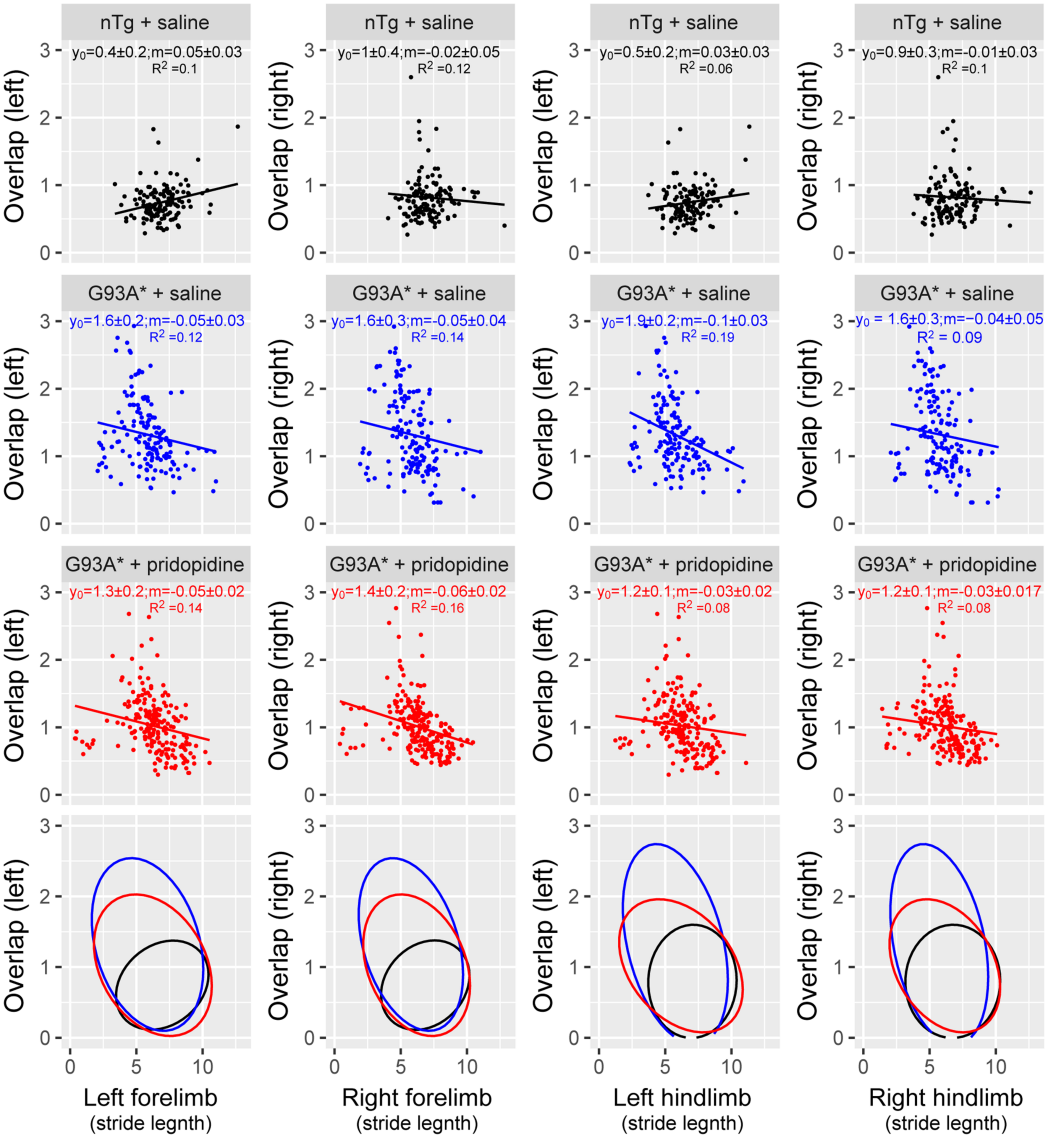
Gait parameter analysis for individual strides. Comparison of left and right overlap with fore and hindlimb stride length reveals changes in correlation between these parameters. **First row:** nTg animals displayed very little correlation between overlap and stride length, implying that healthy animals can coordinate their gait minimizing distance between hind and fore paw step by adjusting fore or hind stride length. **Second row:** In contrast, untreated G93A* animals display an inability to adjust stride length, leading to shorter fore or hind limb strides with larger distances between paw prints (larger overlap). This leads to a negative correlation between the two parameters. **Third row**: Treated G93A* mice showed gait patterns midway between untreated and nTg animals. **Fourth row**: Comparison of nTg, treated and untreated G93A* by quantifying the spread of data by fitting a multivariate ellipse (95% confidence limits) shows a larger beneficial effect of pridopidine treatment on the hind limbs than fore limbs. Data analysed are from nTg + saline (*n* = 15); G93A* + pridopidine (*n* = 23) and G93A* + saline (*n* = 15) mice

The gait data were also analysed by taking all nine gait cycles into account allowing us to look at within cycle correlations and dependencies. In Figure 5 we compared the effect of stride length on the resulting distance to the fore or hind paw imprint. In nTg there is no correlation between the two, since the distance (overlap) is kept at a minimum by adjusting fore or hind limb stride length. Correlation was tested using a generalized estimating equation that takes repeated measures into account. Comparing the standard errors of the slopes shows that nTg mice includes 0, or/and excludes negative values. Confirming that there is no correlation between the two parameters. In contrast, untreated G93A* mice have slopes that are all negative and their confidence intervals exclude 0 and positive values. The treated G93A* mice are intermediate and in the case of left‐overlap vs. left hind limb stride length their slope confidence intervals are different from the untreated G93A* (CI's: −.05, −.01 vs. −.13, −.07, respectively).

The intermediate position of the treated SOD1 mice becomes more apparent if we take the distribution and location of the data points into account and plotting confidence ellipse. The fourth row in Figure 5 shows that hind limb correlations of overlap and stride length are mid‐way between untreated and nTg animals. This is especially surprising, since hind limbs are more affected in SOD1 animals. This shows that the disease modifying effect of pridopidine is more effective on those body regions that are affected earlier.

### 3.4 Pridopidine does not extend the lifespan of G93A* transgenic mice

**FIGURE 6:**
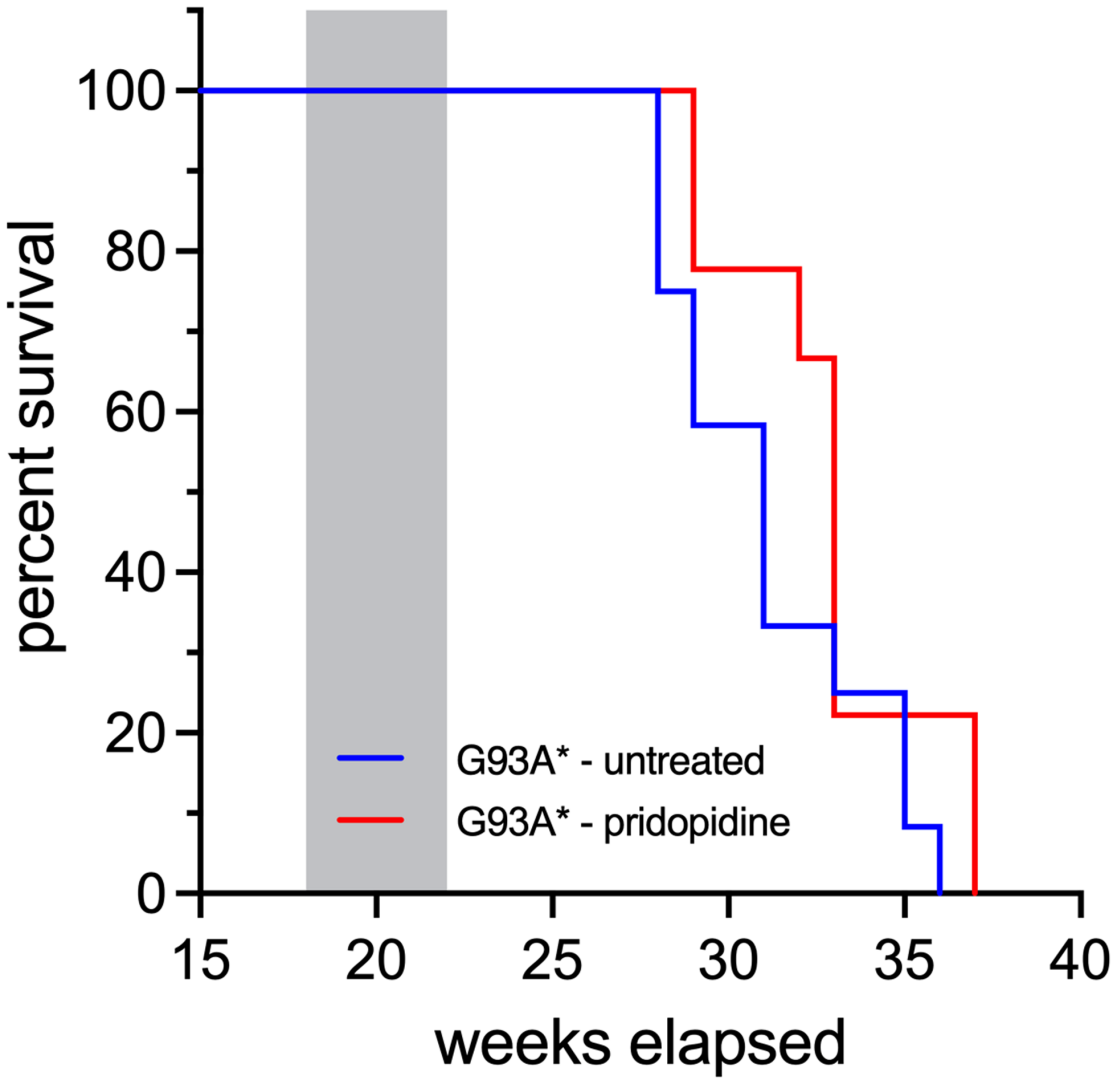
Transient administration of pridopidine does not significantly affect survival in G93A* mice. Kaplan–Meier survival plot to assess changes in survival of G93A* mice after uninterrupted administration of 3.0 mg/kg/day pridopidine for 4 weeks (grey‐shaded area), followed by withdrawal of treatment. Compared to untreated mice (*n* = 13), statistical analysis by the Mantel‐Cox test revealed that temporary pridopidine treatment failed to prolong the survival of G93A* mice (*n* = 9)

Kaplan–Meier survival analysis of the percentage of surviving animals after an uninterrupted administration of 3.0‐mg/kg/day pridopidine compared to non‐treated (saline) for 4 weeks followed by withdrawal of treatment. Pridopidine did not exhibit a statistically significant increase in the lifespan (Mantel–Cox test, *p* > .05) in the median survival of G93A* treated mice (33; IQR 32–33 weeks) compared with the non‐treated mice (31; IQR 29–34 weeks) (Figure 6).

## 4 DISCUSSION

### 4.1 Summary of pridopidine effect

In this study, we evaluated the disease‐modifying effects of the *σ*‐1R, pridopidine, in a delayed onset SOD1 G93A model of ALS (G93A*). Previous studies were conducted in this late‐onset SOD1 model to determine the age at which pridopidine administration was initiated, to coincide with the onset of the first signs of symptoms. Although initiating treatment at an earlier presymptomatic stages of disease may have provided additional protection, we chose to use this time point, as it mimics when patients may be first diagnosed with ALS. Treatment duration was set at 4 weeks to determine whether the hypothesized effects were due to a change in the course of the pathology or a result of symptomatic effects that would disappear after withdrawal of treatment. Our results indicate that the continuous release of pridopidine at 3 mg/kg/day for 4 weeks at pre‐symptomatic stages of disease, impacts the development of symptoms related with the G93A* phenotype, preventing cachexia and improving motor coordination that is maintained up to 5 weeks after treatment withdrawal. Nevertheless, transient administration of pridopidine does not significantly improve survival in G93A* mice.

### 4.2 SOD1 G93A model

G1H murine model is an excellent tool for ALS studies. In this model the high transgene copy number leads to an excess production of human SOD1 protein, which leads to a faster course of disease. (Acevedo‐Arozena et al., 2011) Here we used a delayed onset G93A* mouse model to evaluate disease modifying effects of pridopidine for treatment of ALS. The slower time course of disease progression allows for an extended symptom‐free point of intervention and this slower time course also potentially better reflects human disease. In our study we verified the amount of transgene in our animal cohort and quantified the amount of *hSOD1* ^G93A^ using qPCR. Our results demonstrate that hemizygous G93A* mice exhibit around a 40% loss of transgene. This reduction in transgene is accompanied with an increased average (±SD) survival of 28–33 weeks compared to the average survival of 21–24 weeks for G1H mice. (Gurney et al., 1994) These results, together with the prevention of weight loss in G93A* mice, highlight the importance of using delayed onset ALS models in the development of new therapies. Accelerated models may occlude positive effects by treatments when evaluating body weight and motor behaviour.

### 4.3 Behavioural tests

Selection of the appropriate behavioural tests to evaluate motor deficits is important in the research of new therapeutic strategies for ALS. The Pole Test (Mann & Chesselet, 2015) and Kondziella's Inverted Screen Test (Carter & Shieh, 2015; Miana‐Mena et al., 2005) are commonly used to measure motor coordination in a longitudinal fashion due to their straightforward execution. The inverted screen test measures muscle strength and therefore has been used in ALS research (Deacon, 2013) as a measure of neuromuscular integrity. In this study we found that treatment of G93A* mice with pridopidine prevents the expected loss of body weight, however a direct effect of body weight on animal performance was not observed (Figure 1 and Table 2). Nevertheless, certain behavioural tasks are influenced by body weight and these tests require experimental and control groups to have similar body weights. (Martinez‐Huenchullan et al., 2017) The body weight of our treatment groups was diverse, since male and female mice were grouped together. Further complexities are introduced if the treatment also has an effect on body weight and effects of body weight on animal performance may have been masked, due to the inclusion of males and females. The Pole test measures both hind and fore limb muscle strength and motor coordination. The onset of the disease in G1H mice is marked by muscle weakness in hindlimbs that progress to forelimbs at later stages of disease. (Capitanio et al., 2012; Chiu et al., 1995) In addition, an increase in body weight could make the turning manoeuvre difficult, which is necessary for animals to descend the pole. As a result, this difficulty could be a reason why clear differences were not observed in mice treated with pridopidine in the Pole test T*‐turn* parameter whereas only a significant effect was observed in the T*‐total*.

In order to exclude weight effects and to better distinguish fore and hind limb coordination, we added a third behavioural test and chose to evaluate gait using the Footprint test. In this test, mice with painted paws are allowed to walk freely through a brightly lit narrow alley that leads to a dark box. Our results confirm that this test detects effects of experimental treatments in an ALS model. Gait analysis at 27 weeks of age, revealed an impairment in both hindlimbs and forelimbs affecting motor coordination in transgenic G93A* mice. The disease modifying ability of pridopidine is evident in the partial reversal of these impairments 5 weeks after treatment withdrawal. The treatment effect was stronger in the hindlimbs than the in forelimbs (Figure 5), pointing to a treatment of the earlier affected part of the spinal motoneurons and their neuromuscular junctions.

### 4.4 Sex differences and treatment interaction

Changes in body weight were strongly affected by sex. Linear mixed model analysis revealed sex to be the strongest factor determining body weight variability (see Table 1). Sex affected weight due to nTg males being heavier and also because genotype and treatment affected the weight of the animals. Females treated with pridopidine increased bodyweight similar to that observed in nTg animals. Moreover, motor performance was influenced by sex with males performing worse in both Inverted screen and Pole test (Table 2). Differences between male and female SOD1 G93A (G1H) transgenic mice have been previously described in several studies, reporting that transgenic males mice exhibit a diminished survival, more severe symptoms, and earlier onset compared to age‐matched female G1H mice. (Oliván et al., 2015) Sex differences have also been described in human forms of ALS, (McCombe & Henderson, 2010) with males showing a higher incidence and being affected at a younger age than females.

### 4.5 Mechanisms underlying disease modifying effect

Based on the changes in phenotype that we observe, two potential paths emerge that could underlie the disease modifying effect of pridopidine, either an improved nourishment status based on the diminished cachexia, or a specific neuroprotective effect on the spinal motoneurons.

Cachexia has been associated with ALS (Slowie et al., 1983) and is likely related to the underlying pathology. The evidence that mitochondrial dysfunction and metabolic mechanisms are associated with ALS neurodegeneration is based on observations of an increased pathological accumulation of glycogen in the CNS of SOD1 mice (Dodge et al., 2013) and an increased lipid metabolism in these mice. (Ari et al., 2014) The former could point to hampered metabolism of glucose in neurons and the latter to a switch to lipid metabolism. Nevertheless, how the *SOD1* mutation affects mitochondrial function remains an open issue.

Our findings support the dual/alternative effects via improving nourishment status and providing neuroprotective effects. Pridopidine exerts neuroprotective effects on SOD1^G93A^ motor neurons via activation of the *σ*‐1R (Ionescu et al., 2019b) and multiple downstream pathways: (1) as a chaperone preventing protein misfolding, (Penke et al., 2018) (2) restoring mitochondrial function, (Hayashi & Su, 2007) restoring axonal transport and/or (3) optimizing lipid metabolism. (Hayashi & Su, 2005) Another treatment approach in ALS uses a ketogenic lipid rich diet that has been applied to counter cachexia. Epidemiologic data show that being overweight prolongs survival in these patients. Such a diet has been shown to positively influence survival in animal models of ALS. (Paganoni & Wills, 2013)

*σ*‐1R is a ligand‐operated protein mainly localized in lipid microdomains in endoplasmic reticulum called mitochondria‐associated membranes (MAM). (Penke et al., 2018) Agonist‐mediated activation of specific signalling pathways promotes neuroprotection, synaptogenesis, and has been reported to prevent a decline in memory performance. In addition, changes in sigma‐1 receptor expression and function are implicated in different neurological disorders. (Ryskamp et al., 2019) These characteristics made *σ*‐1R an interesting target and more specifically, their agonists a potential therapeutic tool to combat neurodegenerative disease. It is also important to mention that certain drugs, such as donepezil or fluvoxamine, already approved by the FDA (Ryskamp et al., 2019) to treat specific neurological disorders, were later described as high affinity *σ*‐1R agonists. (Maurice et al., 2006; Nishimura et al., 2008; Ramakrishnan et al., 2014) In the context of ALS, only preclinical studies have focused on *σ*‐1R agonists as novel therapeutics for ALS. (Mancuso et al., 2012; Ono et al., 2014; Ryskamp et al., 2019) One such study used the G1H mouse model of ALS and PRE‐084, a *σ*‐1R agonist that was not pursued further for clinical trials. Improvements in locomotor activity and motoneuron survival were reported in transgenic mice. (Mancuso et al., 2012) The study proposed that *σ*‐1R agonists reduced excitotoxic damage through a modulation of NMDA Ca^2+^ influx and microglial reactivity that resulted in the preservation of motor neurons and neuromuscular junctions. (Mancuso et al., 2012) More recently, others (Ionescu et al., 2019a) confirmed that the S1R agonist pridopidine prevented neuromuscular junction loss, increased survival of motor neurons, and enhanced axonal transport in the G1H model. Our results support and further extends the evidence provided by Ionescu et al., (Ionescu et al., 2019a) to include disease modifying properties by pridopidine. We demonstrate that a 4‐week long delivery of 3.0 mg/kg/day pridopidine prevented weight loss and motor impairments in a delayed onset SOD1 G93A mouse model of ALS.

## 5 CONCLUSION

In summary, pridopidine treatment of G93A* mice for 4‐weeks had a lasting modifying effect on body weight and motor phenotype. These observations were based on statistical analysis that allowed us to compare short and long term pridopidine effects on body weight and confirmed the long‐term effects preventing cachexia. We also used a novel approach to analyse time‐limited motor responses to distinguish effects of sex from genotype treatment interactions during the treatment and post‐treatment phase.

Our limited treatment of 4 weeks, however, failed to extend the survival time of G93A* mice despite preventing cachexia and might be due to disease progression in later affected spinal cord regions. These findings further emphasize the importance of early treatment begin of pridopidine in ALS and point to the need for an extended treatment duration.

## CONFLICT OF INTEREST

The authors declare no conflict of interest.

## AUTHOR CONTRIBUTIONS

H.E.S. and D.M. conceived and designed the research; H.E.S, T.M. and B.L.G. conducted the research; H.E.S, T.M., B.L.G., F.S. and X.L. analysed the data; H.E.S., F.S. and D.M. drafted the manuscript; and H.E.S., T.M., B.L.G., X.L., F.S. and D.M. edited, reviewed and approved the final version of the submitted manuscript.

### PEER REVIEW

The peer review history for this article is available at https://publons.com/publon/10.1111/ejn.15608.

## Supporting information


**Figure S1** Quantitative PCR of the hSOD1 transgene verifies similar levels of expression in the G93A* mice used in the present study in addition to a lower expression of transgene than the G1H founder line. (A) ∆Ct values for hemizygous G93A* mice in the present study were all found to be within a range of 5.537 ± .21 (min. to max.), all falling within the .5 detection limit of this technique. Transgene expression levels were calculated to be ~40% lower than the G1H founder line from The Jackson Laboratory (B6.Cg‐Tg [SOD1*G93A]1Gur/J, stock #: 004435). This lower and uniform transgene expression gives rise to the delayed onset of symptoms and less aggressive progression of disease in the mice evaluated. Each point represents the average ∆Ct value ± SD for each individual animal. Male mice are showed as solid circles while females are exhibited as open circles. (B) A statistical analysis (Student's *t* test) was also performed to evaluate possible differences in average ∆Ct values between males and females for G1H and G93A* mice. No significant sex‐related differences were observed in either G93A* or G1H mice. (C) Addition of ΔCt as a fixed factor to our lmm model of animal weight (excluding nTg animals and genotype as fixed factors) yielded a significant effect (χ^2^ = 14.52, df = 1, *p* < .001). In addition, adding ΔCt x treatment to the model showed a significant interaction (χ^2^ = 22.67, df = 2, p < .001). In (C) we plotted body weight dependence on ΔCt and observe an increase in body weight due to treatment at a range of different ΔCt values.

**Table S1** Randomization and division of animals included in the study and grouped into: nTg controls, saline‐infused G93A* controls, and pridopidine‐treated G93A* groups.

## Data Availability

The datasets acquired during the current study are available from the corresponding author on reasonable request.
