# Supplementary figures and images for: Pridopidine modifies disease phenotype in a SOD1 mouse model of amyotrophic lateral sclerosis
Source: Eur J Neurosci. 2022 Feb 12;55(5):1356–72. doi: 10.1111/ejn.15608 (PMC9305776; doi:10.1111/ejn.15608)

A

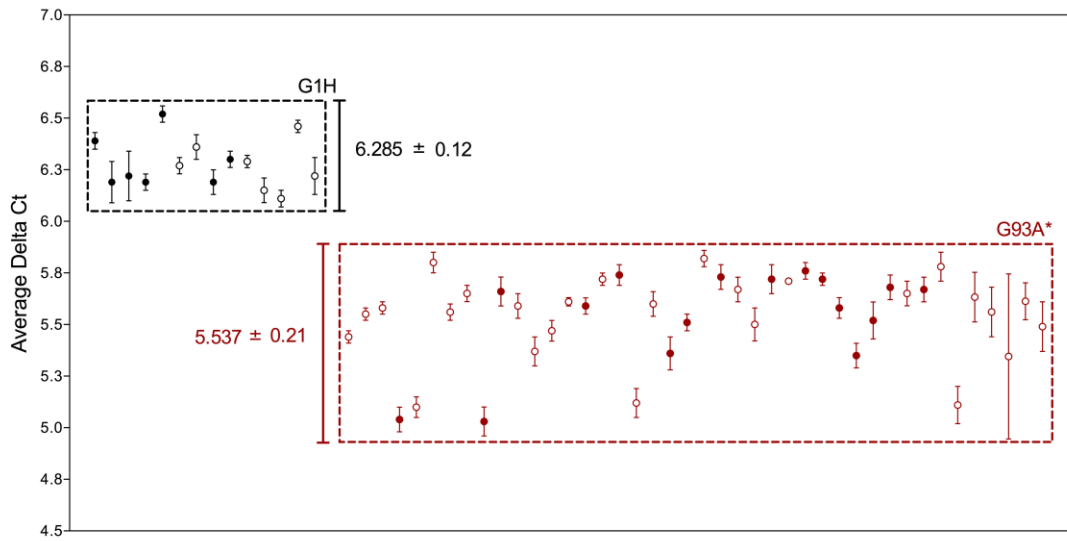

B

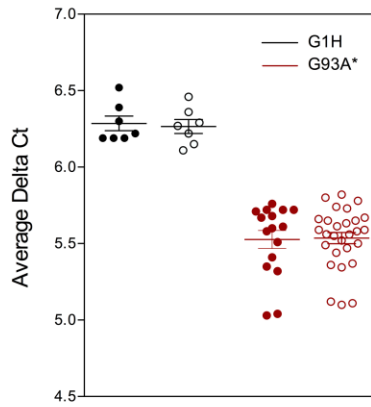

C

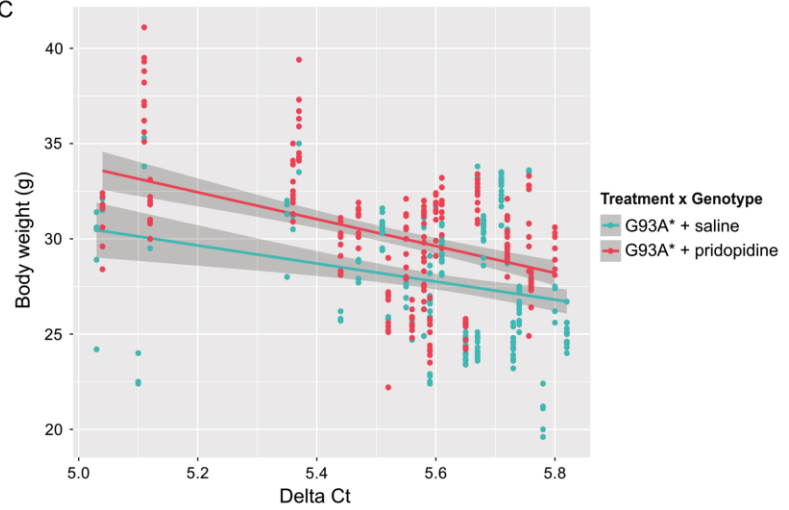

Supplement: Supplementary file 1 — Figure S1 Quantitative PCR of the hSOD1 transgene verifies similar levels of expression in the G93A* mice used in the present study in addition to a lower expression of transgene than the G1H founder line. (A) ∆Ct values for hemizygous G93A* mice in the present study were all found to be within a range of 5.537 ± .21 (min. to max.), all falling within the .5 detection limit of this technique. Transgene expression levels were calculated to be ~40% lower than the G1H founder line from The Jackson Laboratory (B6.Cg‐Tg [SOD1*G93A]1Gur/J, stock #: 004435). This lower and uniform transgene expression gives rise to the delayed onset of symptoms and less aggressive progression of disease in the mice evaluated. Each point represents the average ∆Ct value ± SD for each individual animal. Male mice are showed as solid circles while females are exhibited as open circles. (B) A statistical analysis (Student's t test) was also performed to evaluate possible differences in average ∆Ct values between males and females for G1H and G93A* mice. No significant sex‐related differences were observed in either G93A* or G1H mice. (C) Addition of ΔCt as a fixed factor to our lmm model of animal weight (excluding nTg animals and genotype as fixed factors) yielded a significant effect (χ2 = 14.52, df = 1, p < .001). In addition, adding ΔCt x treatment to the model showed a significant interaction (χ2 = 22.67, df = 2, p < .001). In (C) we plotted body weight dependence on ΔCt and observe an increase in body weight due to treatment at a range of different ΔCt values. [file EJN-55-1356-s002.pdf]
